# Comparison between bidirectional Stratafix^®^ barbed suture and conventional suture in laparoscopic myomectomy: a retrospective study

**DOI:** 10.1186/s12905-020-01030-5

**Published:** 2020-08-05

**Authors:** Kentaro Nakayama, Sultana Razia, Masako Ishikawa, Hitomi Yamashita, Tomoka Ishibashi, Hiroki Sasamori, Kiyoka Sawada, Sonomi Kurose, Seiya Sato, Satoru Kyo

**Affiliations:** Department of Obstetrics and Gynecology, Faculty of Medicine, Shimane University, Enyacho 89-1, Izumo, Shimane 6938501 Japan

**Keywords:** Myoma, Bidirectional barbed suture, Laparoscopic myomectomy

## Abstract

**Background:**

Laparoscopic myomectomy (LM) is one of the techniques feasible for the treatment of intramural myoma. This technique is reported to be difficult when large fibroids are involved because of excessive blood loss during surgery. Skillful and fast suturing appears to be associated with reduced blood loss during LM. In this study we compared the surgical outcomes of using bidirectional Stratafix® barbed suture versus conventional suture during LM.

**Methods:**

This retrospective study included all patients who underwent LM for the treatment of intramural myoma in our institution between 2015 and 2020. The patients were divided into 2 groups according to the technique of suturing during LM: Group 1 comprised patients in whom Stratafix® barbed suture was used (*n* = 29), and group 2 comprised those in whom conventional suture was used (*n* = 15). Data of patient age, myoma size, the number of myoma nodes, hemoglobin levels, total operation time, total suturing time, and blood loss during surgery were compared between the 2 groups.

**Results:**

No significant differences in age (*p* = 0.463) or myoma size (*P* = 0.373) were observed between the 2 groups. Operation time (*P* = 0.0104), suturing time (*P* = 0.007), and blood loss (*P* = 0.0375) during surgery were significantly less with Stratafix® barbed suture than with conventional suture. No patient required intraoperative transfusion or conversion to laparotomy.

**Conclusion:**

The use of bidirectional barbed suture reduces operation time, suturing time, and blood loss. As these new sutures have barbs, no knot-tying is required; thus, continuous suturing becomes very simple and maintaining hemostasis is easy. Unskilled gynecological surgeons who apply this suture technique can also perform LM easily. As the bidirectional barbed suture has multiple points of fixation, this suture technique can reapproximate tissue securely, which reduces the chances of reoperation because of proper suture knotting. Therefore, bidirectional Stratafix® barbed sutures could be an optimal and efficient alternative to conventional sutures for use by gynecological surgeons in Japan.

## Background

Uterine myomas, also known as leiomyomas or fibroids, are very common in women of childbearing age. Intramural myomas are the most frequent (58–79%) among all the observable uterine myomas [1, 2]. The quality of life can decrease as a result of myomas due to menorrhagia, dysmenorrhea, and pelvic pain. Several studies established that there are various advantages of laparoscopic myomectomy (LM) over the laparotomic and minilaparotomic approaches for the treatment of uterine myomas, including shorter hospital stay, less postoperative pain, faster recovery, and lower intraoperative hemoglobin drop [3–7]. However, LM has been the subject of many controversies because of excessive blood loss, prolonged operation time, postoperative complications, and prolonged hospital stay, especially when multiple myomas are involved [8]. Many new methods were introduced for reducing bleeding during myomectomy such as ligation of uterine artery, oxytocin use, and injection of vasoconstrictor agents [9–11]; however, excessive hemorrhage during myomectomy remains a major challenge for the gynecologic surgeon. With a fast suturing technique, the myometrium remains open for less time, thereby reducing intraoperative bleeding during myomectomy.

Suturing and knot-tying are challenging laparoscopic skills that require extensive training. Barbed suture has been recently introduced to facilitate laparoscopic suturing. A suture with bidirectional barbs offers several advantages over conventional sutures: 1) It is self-anchoring and is balanced by the countervailing barbs, and hence, no knots are required. 2) It self-anchors every 1 mm of tissue, yielding more consistent wound opposition; this may result in a more “watertight” seal. 3) Because it is knotless, it can securely re-approximate tissues in less time, at less cost, and with less aggravation [12, 13]. Pierluigi et al. found that the mean operation time was shorter and intraoperative bleeding volume was less with Stratafix barbed sutures than conventional sutures in laparoscopic posterior myomectomy [14]. The efficacy and safety of barbed suture have been demonstrated in various gynecologic surgeries in many countries; however, to our knowledge, no comparable studies have been conducted in Japan. Therefore, the aim of this study was to compare a bidirectional barbed suture (Stratafix®, Ethicon Inc., USA) with conventional suture (Vicryl®, Ethicon Inc., USA) during LM with respect to the surgical outcomes. To our knowledge, this is the first report on the use of Stratafix, a bidirectional barbed suture, during LM in Japan.

## Methods

**Fig. 1:**
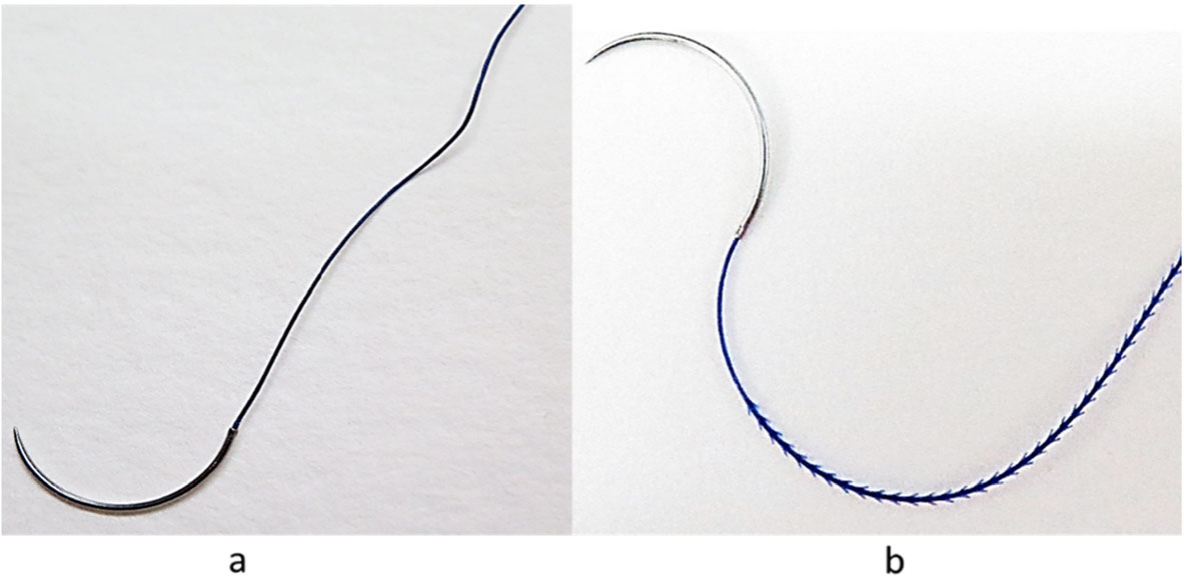
**a** Vicryl® conventional suture (Ethicon®, USA). **b** Bidirectional Stratafix® barbed suture with barbs (Ethicon®, USA)

This retrospective study included 44 patients who underwent LM for benign uterine leiomyomas at our institution between April 2015 and June 2020. The inclusion criteria were a diagnosis of intramural myomas with the largest diameter measuring between 5 and 13 cm and less than three myoma nodes. Hypermenorrhea and dysmenorrhea were the indications for operation. The patients were divided into two groups according to the method of suturing; the patients who underwent LM using Stratafix^®^ barbed suture (group 1, *n* = 29) and those in whom a conventional control suture technique was used (group 2, *n* = 15) (Fig. 1). Continuous suturing in two or three layers was performed in both groups.

The main outcome measures chosen for the current analysis were total operation time, total suturing time, estimated blood loss during surgery, and changes in hemoglobin level at 1 day post operation. Estimated blood loss during surgery was measured by suction volume. All patients provided written informed consent for the procedure of laparoscopic surgery. This work was approved by the Institutional Review Board, Shimane University (IRB No. 201912120–1).

Data collected from the hospital database included age, body mass index (BMI), previous surgeries, preoperative symptoms, operation time, blood loss, length of hospital stay, uterine weight on pathological examination, and follow-up.

### Statistical analysis

The data were compared between two groups using student t tests. *P*-value of less than 0.05 was considered statistically significant. Statistical analysis was performed using SPSS statistical software, version 21 (SPSS, Inc., Chicago, IL, USA).

## Results

**Fig. 2:**
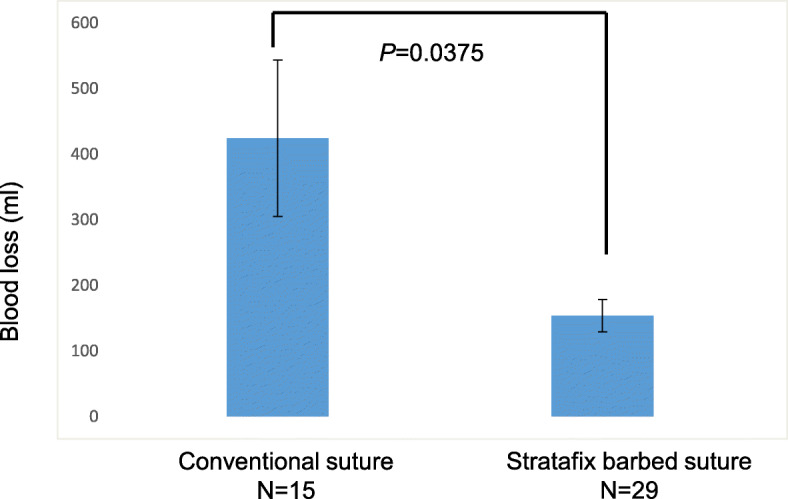
Differences between the conventional suture group and Stratafix suture group with respect to blood loss*. P* values were obtained by Student’s t test

**Fig. 3:**
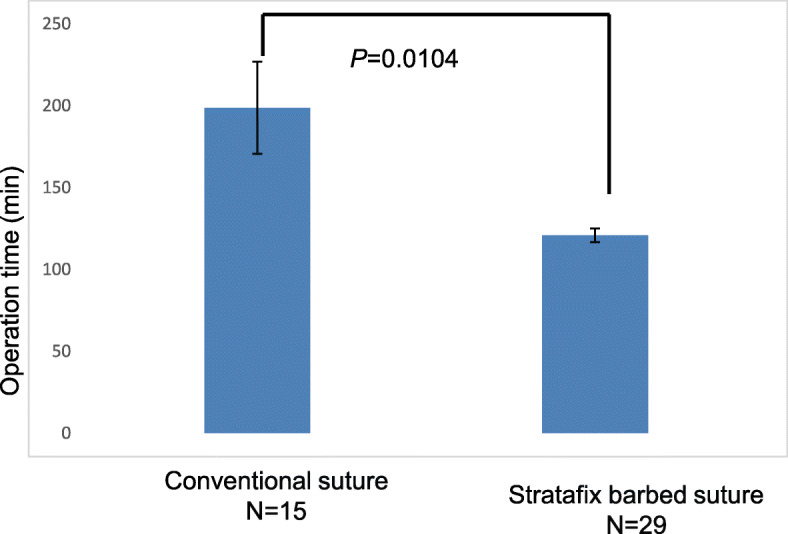
Differences between the conventional suture group and Stratafix suture group with respect to operation time*. P* values were obtained by Student’s t test

**Fig. 4:**
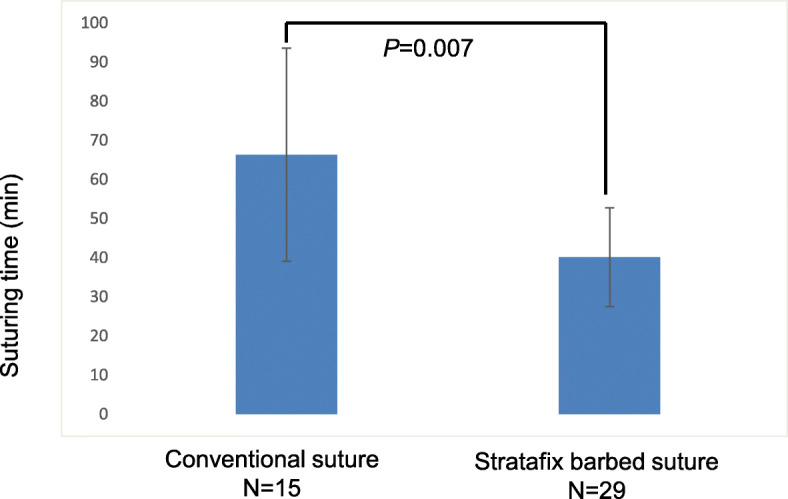
Differences between the conventional suture group and Stratafix suture group with respect to total suturing time*. P* values were obtained by Student’s t test

A total of 44 patients who underwent LM for intramural myomas during the study period were included. No significant differences in age (38 ± 4, group 1 vs. 40 ± 3, group 2, *P* = 0.463), BMI (20.8 ± 1.7, group 1 vs. 22.1 ± 3.3, group 2, *P* = 0.083), the number of myomas (1.67 ± 1.3, group 1 vs. 1.53 ± 1.5, group 2, *P* = 0.653), and maximum myoma size (7.4 ± 2.5, group 1 vs. 8.6 ± 2.6, group 2, *P* = 0.373) were noted between the 2 groups (group 1; barbed suture vs. group 2; conventional suture). The median operation time and blood loss were significantly less in group 1 (120 min, 154 mL) than in group 2 (198 min, 424 mL, respectively) (Fig. 2 and Fig. 3). The suturing time in group 1 was significantly shorter than that of group 2 (40.1 ± 12.6 min, group 1 vs. 66.2 ± 27.2 min, group 2, *P* = 0.007) (Fig. 4). There was no significant difference in the change of hemoglobin levels 1 day after operation between the two groups (1.12 ± 0.8 g/dL, group 1 and 1.55 ± 0.7 g/dL, group 2, *P* = 0.357). There was no significant difference in the postoperative hospital stay between the two groups (group 1; 4.5 ± 1.9 days and group 2; 4.7 ± 1.8 days, *P* = 0.562). No intraoperative or postoperative complications including paralytic ileus occurred in patients of either group. Surgical pathology confirmed the diagnosis of intramural myomas in all cases.

## Discussion

LM is one of the accepted and preferred methods for the treatment of intramural myoma, especially in patients who desire to continue their fertility or intend to preserve their uterus [15]. LM is a controversial procedure, although it is now considered feasible [16]. The technique is reported to be difficult, time consuming, and has a high risk when large fibroids are involved due to increased intraoperative blood loss during dissection. Over the past few years, several new methods have been introduced to minimize bleeding during myomectomy [9–11]. Skillful as well as fast laparoscopic suturing is also a significant factor that influences intraoperative uterine bleeding [17, 18].

In recent years, a self-anchoring, bidirectional barbed suture that does not require knot-tying was developed for laparoscopic surgery. The Stratafix® barbed suture without knot-tying has changed the laparoscopic suturing procedure and reduced operation time. Our study showed that a significantly lower operation time as well as blood loss was observed with the bidirectional Stratafix barbed suture during LM than with conventional suture. The suturing technique utilizing Stratafix® sutures was found to reduce operation time by approximately 39% and blood loss by 63.6% when compared to conventional suturing. Pierluigi et al. [14] found that operation time decreased by 9.5% and blood loss by 10.7% with Stratafix® barbed suture compared to Vicryl® suture. Aoki et al. [19] observed that a suturing technique applying V-Loc® barbed suture (Covidien, Mansfield, MA) materials reduced the operation time of LM by approximately 25% when compared to conventional suture. Several studies, including a randomized trial using unidirectional barbed suture versus continuous suture on the effectiveness of barbed suture have concluded that barbed sutures decrease operation time and intraoperative bleeding [12, 20–22]. A possible reason for the reduced operation time using a barbed suture is that because of the barbs, once the suture has been pulled taut, the points of commissure will not loosen even if the assistant does not maintain tension on the suture thread.

Our study included a small number of patients in each group, thereby making it difficult to draw a clear-cut conclusion about the findings. Therefore, further investigation with a larger study population is required. Moreover, our study is retrospective in nature and the technique was evaluated in one medical hospital by only one surgeon; therefore, it may be difficult to extrapolate our findings. Consequently, further randomized control trials are necessary.

To our knowledge, this is the first report in the Asian region that has compared the surgical outcomes of bidirectional Stratafix® barbed sutures versus conventional sutures, and this is the most significant strength of this study. Our study demonstrated that the use of Stratafix® barbed suture for LM significantly reduces operation time, suturing time, and blood loss.

## Conclusion

Stratafix, a bidirectional barbed suture, can shorten operation time, suturing time and blood loss during LM. This new suture has barbs that maintain tensile strength evenly along the total length of the wound without knots. Therefore, continuous suturing becomes simple and maintaining hemostasis is easy. Moreover, gynecological surgeons who are not well versed with the technique of suturing can easily perform LM by applying this technique. On the basis of this report, bidirectional barbed sutures could be an optimal and efficient alternative to conventional sutures to assist gynecological surgeons in performing LM. Widespread adoption of this technique in Japan is recommended.

## Data Availability

Data to replicate findings are in the Figures of the main paper. Due to patient privacy protection, any additional materials of the study are only available upon individual request directed to the corresponding author.

## Abbreviations

BMI: Body mass index
LM: Laparoscopic myomectomy
